# Dengue in Singapore from 2004 to 2016: Cyclical Epidemic Patterns Dominated by Serotypes 1 and 2

**DOI:** 10.4269/ajtmh.17-0819

**Published:** 2018-05-29

**Authors:** Jayanthi Rajarethinam, Li-Wei Ang, Janet Ong, Joyce Ycasas, Hapuarachchige Chanditha Hapuarachchi, Grace Yap, Chee-Seng Chong, Yee-Ling Lai, Jeffery Cutter, Derek Ho, Vernon Lee, Lee-Ching Ng

**Affiliations:** 1 Environmental Health Institute, National Environment Agency, Singapore, Singapore; 2 Public Health Group, Ministry of Health, Singapore, Singapore; 3 Environmental Public Health Division, National Environment Agency, Singapore, Singapore; 4 School of Biological Sciences, Nanyang Technological University, Singapore

## Abstract

Singapore has experienced periodic dengue epidemics despite maintaining a low *Aedes* house index. Each epidemic was associated with a switch in the predominant serotype. We investigated the temporal dynamics of dengue fever and dengue virus (DENV) and analyzed the epidemiological and entomological patterns of dengue in Singapore from 2004 to 2016. The case surveillance is based on a mandatory notification system that requires all medical practitioners to report clinically suspected and laboratory-confirmed cases. Circulating (DENV) serotypes are monitored through a virus surveillance program. Entomological surveillance involves inspections for larval breeding and monitoring of adults using gravitraps. Singapore experienced a similar epidemic pattern during 2004–2007 and 2013–2016. The pattern involved a 2-year DENV-1 epidemic occurring after a switch in the predominant serotype from DENV-2 to DENV-1, followed by a “lull” year. Thereafter, the predominant serotype switched back to DENV-2, tailed by a small-scale epidemic. Across the years, the highest incidence group was in the 25–44 years age group. The incidence rate of those aged ≥ 55 years was about half of that of the 15–24 years age group during DENV-1 predominant years. However, it was almost equal to the younger age group in DENV-2 predominant years. Types of *Aedes aegypti* breeding habitats remained similar. Dengue incidence was significantly higher in areas with high breeding percentage (BP) than areas with low BP (*P* < 0.05). In conclusion, the oscillation of DENV-1 and DENV-2, throughout the 13-year period, led to a cyclical epidemic pattern and older adults were more affected by DENV-2 than DENV-1.

## INTRODUCTION

The global incidence of dengue has increased drastically over the past 50 years. It was estimated that 390 million (95% credible interval 284–528) dengue infections occur worldwide per year, including 96 million (67–136) apparent manifestations in 2010,^1^ and Asia-Pacific countries bear about three-quarters of the global dengue disease burden.^2^ In Singapore, dengue is endemic because of the presence of vectors of dengue virus (DENV), *Aedes aegypti* and *Aedes albopictus* mosquitoes, and conducive environmental conditions required for virus transmission. The first outbreak of dengue fever (DF) in Singapore was reported in 1901.^3^ An outbreak of dengue hemorrhagic fever (DHF) involving 70 hospitalized cases was recorded in 1960.^4,5^ Following then, large epidemics occurred almost every year in the 1960s, affecting mostly the pediatric age group.^6–9^

A comprehensive nationwide *Aedes* prevention and control program was launched in Singapore in 1969 and fully implemented in 1973. This incorporated source reduction, health education, and law enforcement.^9^ Since the 1960s, the *Aedes* house index (HI) (percentage of residential premises found to be breeding *Aedes* mosquitoes) decreased sharply from 48% in 1966 to 13% in 1973 and has been further suppressed to 0.7% by 2016.^10^ The dengue incidence decreased from 42.2 per 100,000 population in 1969 to 3–10 by 1973^9–11^ and remained low at eight cases per 100,000 population for about a decade.^12^ However, since the late 1980s, dengue started to resurge in a typical 5- to 6-year epidemic cycle, although the *Aedes* HI remained less than 1%.^10^ A low herd immunity of the Singapore resident population was hypothesized to have facilitated the epidemics, a paradoxical situation arising from the country’s longstanding and intensive vector control program.^13^

To understand the recent epidemiology of dengue in Singapore, we investigated the temporal pattern of DF and DENV dynamics in Singapore from 2004 to 2016 and analyzed the epidemiological and entomological data collected through the dengue surveillance program.

## MATERIALS AND METHODS

### Case data collection.

In Singapore, the Ministry of Health (MOH) is responsible for the epidemiology and clinical management of dengue, whereas the National Environment Agency (NEA), an agency under the Ministry of Environment and Water Resources, is responsible for vector surveillance and control. Under the Infectious Diseases Act, it is mandatory for all medical practitioners and clinical laboratories to notify MOH of all clinically suspected and laboratory-confirmed dengue cases within 24 hours of diagnosis. Laboratory confirmation of dengue cases is achieved through nonstructural protein 1 (NS1) antigen detection, viral RNA detection by polymerase chain reaction (PCR), or immunoglobulin M detection.^14,15^ Imported dengue cases are defined as cases who have traveled to a dengue-endemic area outside of Singapore within 7 days before the onset of illness. National Environment Agency’s epidemiologically trained officers interview the cases when necessary to obtain epidemiological and demographic data including occupation, residential and school/workplace addresses, and dates of diagnosis and onset of illness. Data on deaths from DF/DHF are obtained from the national Registry of Births and Deaths.

### Virus data collection.

Virus data are collected via a virus surveillance system previously described.^16^ The national virus surveillance program has, since 2013, expanded from a network of private general practitioners and public acute-care hospitals to include samples from government primary care clinics and, since 2015, to private laboratories that serve private hospitals and clinics. Residual samples tested positive for DENV by either NS1 antigen detection assays (the most commonly used test) or PCR are further analyzed by PCR^14,17,18^ to determine the serotype at the Environmental Health Institute (EHI) of NEA and the National Public Health Laboratory (NPHL) of MOH. Environmental Health Institute and NPHL both used the same reverse transcription polymerase chain reaction assay and quality assurance audits are regularly carried out in the laboratories. The laboratory serotyping coverage of dengue cases has increased from 13% in 2005 to more than one-third of all reported cases in 2016.

### Vector data collection.

Vector data are collected through an inspection regimen conducted by 800 NEA vector control officers to identify mosquito breeding at homes and other premises. Such source reduction and vector surveillance efforts are enhanced in areas with high risk of *Ae. aegypti* breeding and dengue transmission. Since 2013, manpower has been dedicated to high-risk areas such as construction sites and areas with prolonged dengue transmission. Mosquito immatures (larvae and pupae) collected are identified up to the species level at EHI. The data collected are used to identify key breeding habitats of *Aedes* mosquitoes and to determine the spatial distribution of *Ae. aegypti* and *Ae. albopictus*. The low *Aedes* HI, together with the presence of cryptic sites, has rendered HI insensitive for gauging *Ae. aegypti* population in the community. As such, since mid-August 2013, adult *Aedes* population monitoring was conducted weekly with 3,000 gravitraps^19^ at 34 sentinel sites.

### Vector control.

A cluster of notified cases suggests active, localized transmission of dengue. Enhanced vector control is performed in clusters as defined by 1) two or more cases epidemiologically linked by place (residential or workplace/school), 2) cases occurring within a radius of 150 m from the index and from all subsequent cases, and 3) cases with their onset of illness within a 14-day period. On top of source reduction, cluster management includes other methods such as indoor space spraying, aerosol cans, ultra-low–volume treatment,^20^ and outdoor thermal fogging where necessary.

### Data analysis.

All laboratory-confirmed dengue cases reported to MOH were included in the analysis. Incidence rates (IRs) and incidence rate ratios (IRRs) of DF and DHF cases were calculated based on case notifications and the mid-year total population estimates obtained from the Department of Statistics, Singapore.^21^ Estimates of Singapore resident population (comprising Singapore citizens and permanent residents) were used for computation of IRs by ethnic group and type of residential premises; all other IRs were calculated using the estimates for total population. The Wilcoxon rank-sum test and Kruskal–Wallis test, where appropriate, were used to test for group differences in IRs using R software version 3.0.2.^22^

Mosquito surveillance data collected by vector control operations and adult mosquitoes caught in gravitraps were used to calculate the *Ae. aegypti* breeding percentage (BP) and gravitrap *Ae. aegypti* index (GI_aeg_), respectively. *Aedes aegypti* BP was defined as the proportion of *Ae. aegypti*–positive breeding sites of the total number of *Aedes*-positive breeding sites (*Ae. aegypti* and *Ae. albopictus*) per year in a defined area.^23^ Gravitrap *Ae. aegypti* index expresses the percentage of gravitraps that caught at least one *Ae. aegypti* mosquito in a particular location. Breeding percentage for each residential subzone was calculated using the spatial join tool in the analyst tool of ArcGIS version 10.4 Arcmap software (ESRI, Redlands, CA).^24^ Breeding percentage values were classified according to three classes determined through receiver operating characteristic curve analysis on *Aedes* mosquito surveillance data collected from 2003 to 2013: low BP (≤ 20%), medium BP (21–40%), and high BP (> 40%) (Environmental Health Institute, unpublished data). Gravitrap *Ae. aegypti* index values were classified according to low GI_aeg_ (< 12%) and high GI_aeg_ (≥ 12%) based on the computation of odds ratios using data collected from gravitraps in active dengue clusters from 2012 to 2013 (Environmental Health Institute, unpublished data). Pearson’s correlation coefficient was calculated between the dengue incidence and mean GI_aeg_ from 2013 to 2016. The spatial distribution of dengue cases was generated using the kernel density tool in the spatial analyst toolbox of ArcGIS version 10.4 ArcMap software based on a search radius of 400 m. Statistical significance was taken as *P* < 0.05.

## RESULTS

### Temporal pattern of dengue epidemics and predominant serotypes.

**Figure 1.**
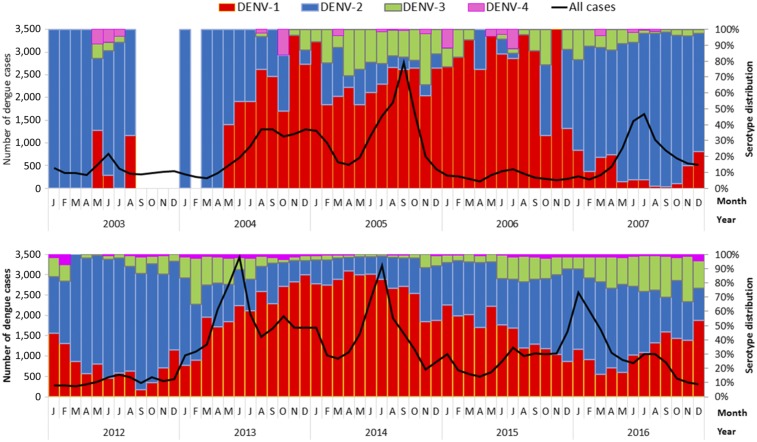
Monthly number of all dengue cases and distribution by serotype based on virus surveillance program, 2003–2016.

Singapore experienced a similar epidemic pattern from 2004 to 2007 (the first epidemic cycle in the new millennium) and from 2013 to 2016 (second cycle). The two epidemics shared several common features (Figure 1): 1) 2-year epidemics during the 2004–2005 and 2013–2014 periods, associated with a switch in the predominant serotype from DENV-2 to DENV-1; 2) monthly cases in the first year of each epidemic cycle (2004 and 2013) peaked during the traditional dengue season from June to September, but remained at an elevated level by year end, a period when dengue situation would usually subside otherwise; 3) the third year of each cycle (2006 and 2015) saw a drastic drop in dengue cases; 4) in the fourth year, the dominant serotype switched back to DENV-2, although the switch was less pronounced until late 2016; and 5) the average IR of the DENV-1 predominant epidemics (2004–2005 and 2013–2014) in each of the two cycles was about 1.5 times that of the ensuing 1-year DENV-2 predominant epidemics in the two cycles, respectively. The proportion of DENV-3 and DENV-4 cases remained low during both cycles, although a rise in DENV-3 was evident in both cycles, whenever there was a peak in DENV-1 cases.

### Epidemiological findings.

The highest proportion of DHF cases (2.7%) was reported during 2004–2005 and remained less than 1% since 2010. The highest case fatality rate was recorded in 2006 (0.3%) and remained at an average of 0.05% from 2012 to 2016 (Table 1). The median age of dengue deaths was 58 years (range: 1–95 years).

**Table 1:** Number of reported dengue cases, proportion of DHF, number of dengue deaths, and CFR, 2004–2016

Year	Number of dengue cases	Proportion of DHF cases (%)	Number of deaths	CFR (%)
2004	9,459	1.8	9	0.10
2005	14,209	2.7	25	0.18
2006	3,127	2.4	10	0.32
2007	8,826	2.1	24	0.27
2008	7,031	1.2	10	0.14
2009	4,497	1.0	8	0.18
2010	5,363	0.6	6	0.11
2011	5,330	0.4	6	0.11
2012	4,632	0.7	2	0.04
2013	22,170	0.4	8	0.04
2014	18,326	0.1	6	0.03
2015	11,294	0.1	6	0.05
2016	13,085	0.2	12	0.09

CFR = case fatality rate; DHF = dengue hemorrhagic fever.

Adults were more affected by dengue infection, with the highest IR (Table 2) in the 25–44 years age group. However, the proportion of notified cases in the age group ≥ 55 years was significantly higher in DENV-2 predominant years (17.9%) when compared with DENV-1 predominant years (12.8%) (*P* < 0.05) (Table 3). Although the IR of ≥ 55 years age group was only about half of that of the 15–24 years age group during the DENV-1 predominant years, it was similar to that of the younger age group in DENV-2 predominant years (Table 3).

**Table 2:** Incidence of reported indigenous dengue cases per 100,000 population by predominating serotype, gender, age group, and ethnic group, 2004–2016

	Year												
	2004*	2005*	2006	2007*	2008	2009	2010	2011	2012	2013*	2014*	2015	2016
Predominant serotype	DENV-1	DENV-1	DENV-1	DENV-2	DENV-2	DENV-2	DENV-2	DENV-2	DENV-2	DENV-1	DENV-1	DENV-1	DENV-2
All	223.1	328.9	64.7	180.6	137.0	83.9	98.1	98.4	82.2	404.9	325.6	196.1	229.1
Gender													
Male	271.9	374.7	76.0	209.3	161.0	98.1	114.6	112.2	98.5	494.5	408.3	235.8	262.3
Female	173.4	282.2	53.1	150.3	111.0	68.5	80.2	83.4	64.6	306.7	234.8	152.8	193.1
Age group (year)													
0–4	48.6	86.9	18.3	48.0	43.6	22.6	29.5	32.0	17.0	70.1	80.5	42.1	48.5
5–14	190.9	334.3	43.1	102.6	76.8	43.1	64.7	66.2	42.8	262.0	244.2	132.3	163.4
15–24	326.2	474.3	67.7	176.7	145.7	97.9	105.5	105.9	91.0	527.2	415.3	238.4	275.7
25–34	284.7	376.8	67.8	188.8	152.8	90.6	105.6	100.3	91.7	483.8	399.9	232.3	263.3
35–44	247.5	362.8	68.5	219.7	164.4	91.8	132.8	112.3	97.5	476.4	389.2	221.2	264.4
45–54	175.1	262.1	53.0	174.4	120.4	77.1	94.0	102.7	77.3	392.2	321.3	201.7	246.2
55+	138.5	217.7	94.4	228.9	156.9	100.5	85.3	105.4	87.2	297.9	205.8	158.8	182.1
Ethnic group													
Residents	207.3	309.6	58.6	166.5	114.7	75.8	83.7	94.4	300.1	351.5	272.2	185.3	218.8
Chinese	220.3	322.3	58.7	174.8	122.6	79.3	87.9	99.8	787.1	361.8	280.3	190.3	221.3
Malay	169.9	300.0	58.0	136.8	82.8	56.6	61.7	81.3	59.3	335.4	248.1	146.7	171.7
Indian	128.7	178.3	40.6	113.0	71.1	55.6	54.0	55.3	527.1	195.1	141.1	106.8	126.4
Others	308.2	445.1	126.7	264.9	197.5	130.8	161.4	134.9	115.4	618.2	554.1	449.5	614.3
Nonresidents	294.7	413.0	89.6	230.9	205.0	108.3	139.5	109.2	100.9	537.1	454.9	222.0	253.4

DENV = dengue virus.

* Dengue epidemic years. The age group with the highest incidence rate is highlighted in gray.

**Table 3:** Age group distribution and IR of reported indigenous dengue cases per 100,000 population aggregated across DENV-1 and DENV-2 predominant years

	DENV-1 predominant years		DENV-2 predominant years	
	2004–2006 and 2013–2015		2007–2012 and 2016	
Age group (year)	Proportion of dengue cases (%)	IR	Proportion of dengue cases (%)	IR
0–4	1.0	58.0	1.1	34.4
5–14	7.8	200.8	5.9	79.3
15–24	19.1	344.9	16.4	140.9
25–34	26.1	317.3	24.0	142.7
35–44	20.4	301.1	21.0	155.3
45–54	12.7	240.4	13.6	128.0
55+	12.8	192.1	17.9	134.4

DENV = dengue virus; IR = incidence rate.

The trends of indigenous dengue cases by gender and ethnic group remained similar from 2004 to 2016. Men consistently constituted a higher proportion of dengue cases (60%) compared with women, but there was no significant difference in the IR between men and women across all years. There was a significant difference in the IR between the ethnicities across all years (*P* < 0.05). Singapore residents of Chinese ethnicity had the highest IR, followed by Malays and Indians, across all years. The IR between nonresidents and residents was not significantly different with the IRR ranging from 1.2 to 1.8 (Table 2).

**Figure 2.**
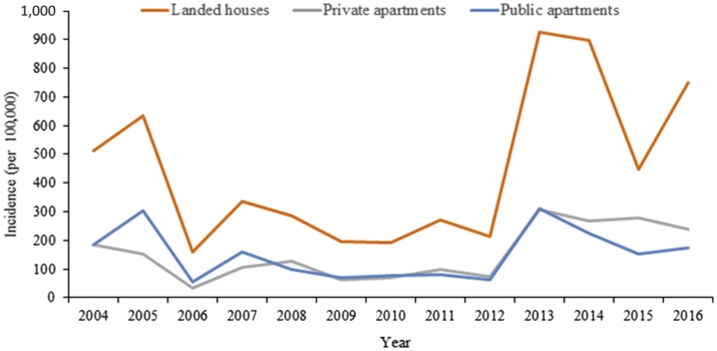
Dengue incidence rate of reported cases among Singapore residents by types of residential premises, 2004–2016.

Type of residential premises was another demographic trait associated with dengue. Of the indigenous cases among Singapore residents, the highest IR was consistently among those living in landed houses compared with those living in high-rise apartments. Since 2013, there was a steady increase in the IR of those staying in private apartments (Figure 2). On the other hand, the proportion of indigenous cases reported in public apartments decreased from 81.3% in 2005 to 66.1% in 2015.

The average proportion of clustered cases increased from 35.3% of all cases during the first epidemic cycle to 51.8% in the second cycle. In parallel, the total number of clusters also increased by 89.5% (Supplemental Figure 1).

The proportion of imported cases fluctuated between 1.2% and 9.1% from 2004 to 2016. The proportion has remained less than 4% since 2013. The majority (> 75%) of the imported cases were from Southeast Asian countries.

### Entomological findings.

*Aedes aegypti* constituted 55% of the *Aedes* mosquito breeding detected in and around residential homes from 2004 to 2016. The major breeding habitats for both *Ae. aegypti* and *Ae. albopictus* were domestic containers, ornamental containers, flower pot plates/trays, and discarded receptacles (Supplemental Figure 2). *Aedes aegypti* inhabited urban areas, whereas *Ae. albopictus* was ubiquitous but in higher proportion in less urban areas with greenery. Dengue incidence was significantly higher in urban areas with high *Ae. aegypti* BP compared with areas with low *Ae. aegypti* BP (*P* < 0.05) (Supplemental Figure 3). There was no significant correlation (*r* = 0.18) between dengue incidence and mean GI_aeg_ from E-week 34 of 2013 to E-week 33 of 2016 (*P* > 0.05), although the site-specific analysis showed higher odds of exceeding the median dengue incidence in high GI_aeg_ areas (≥ 12%) than low GI_aeg_ (< 12%) areas (odds ratio = 8.7, *P* < 0.05).

## DISCUSSION

The epidemiology of dengue in Singapore over the last decade showed some interesting trends. Although all four DENV serotypes have been endemic in Singapore since they were monitored in 1980s,^5^ the predominant serotypes associated with epidemics in the last decade have oscillated between DENV-1 and DENV-2, whereas the proportions of cases due to DENV-3 and DENV-4 have remained low. Dengue virus-3 was last dominant in 1998.^25^ Since then, it has been involved only in occasional, small localized outbreaks, most recently in 2016 in southeast Singapore.^26,27^ Dengue virus-4 cases have generally been sporadic. This low proportion of DENV-3 and DENV-4 cases detected from the DENV surveillance program is consistent with the lower prevalence of DENV-3 and DENV-4 neutralizing antibodies, when compared with DENV-1 and DENV-2, in the local population. Among the various age groups in 2009, the prevalence of DENV-3 neutralizing antibodies was 2.7–57.7% and that of DENV-4 neutralizing antibodies was 2.1–31.8%, significantly lower than the 9.7–77.9% for DENV-1 and 14.0–86.6% for DENV-2.^28^ Because the blocking of DENV-1 and DENV-2 transmission by *Wolbachia* wMEL is limited,^29,30^ the dominance of DENV-1 and DENV-2 in Singapore therefore has an implication on the possible approach of replacing local mosquitoes with *Wolbachia*-infected *Aedes* for dengue control, as considered by other countries. The dominance of DENV-1 and DENV-2 is also expected to limit the effectiveness of the only approved tetravalent dengue vaccine, Dengvaxia^®^, in Singapore, as the vaccine has yielded lower overall efficacy against DENV-1 and DENV-2 in clinical trials.^31,32^

The low incidence of infections associated with DENV-3 and DENV-4 in Singapore contrasted with their more frequent involvement in outbreaks in the Southeast Asian region in the past 20 years. Thailand and the Philippines, where these two serotypes were historically first recorded, have regularly reported the epidemic transmission of DENV-3 and DENV-4.^33^ Between 1999 and 2002, Thailand saw the most frequent occurrence of DENV-3 and DENV-4 cases, when compared with the other Southeast Asian countries.^33^ Dengue virus-3 and DENV-4 were also the dominant serotypes in Jakarta, Indonesia, in 2004^34^ and in Malaysia in 2012.^35^ Interestingly, in Bangkok, epidemics oscillated between DENV-1, DENV-2, and DENV-3, with an isolated epidemic of DENV-4.^34^ In Singapore, the smaller numbers of DENV-3 and DENV-4 cases, despite the low prevalence of the respective antibodies in the local population, are not well understood. It is possible that DENV-3 and DENV-4 have a weaker compatibility to Singapore’s *Ae. aegypti* mosquitoes and, thus, have lower epidemic potential when compared with DENV-1 and DENV-2. Alternatively, it could be because of protective antibodies that were not measured by plaque reduction neutralization tests. The complexity of community immunity to different serotypes has been demonstrated by cluster studies in Thailand showing that the levels of neutralizing antibody titers required for protection from the disease varied for each of the four DENV serotypes.^36^ These results are also supported by the outcome of phase 2b and 3 trials of the dengue vaccine, Dengvaxia, which has also shown discordance between the presence of neutralizing antibodies against each of the four DENV serotypes with protection from infection.^37–39^ In addition, seroprevalance data may not accurately reflect the actual infecting serotypes because of cross-reactivity in the serological assay. Another significant finding from this study is the increased risk of dengue in the older population during DENV-2 outbreaks. Dengue virus-2 is often associated with secondary infections as compared with other serotypes and is also associated with the greatest percentage of severe cases among secondary infections.^40–44^ Considering that the seroprevalence of Singapore’s population was 16% in the 16–20 years age group and more than 85% among those aged ≥ 55 years,^28^ it is likely that a substantial number of older adults were susceptible to a secondary dengue infection, which was more apparent when caused by DENV-2. This postulation is consistent with findings from several studies. An 18-year interval study between a DENV-1 outbreak in 1977–1979 and a DENV-2 outbreak in 1997 in Santiago de Cuba reported that secondary infections of DENV-2 and DENV-4 serotypes resulted in increased disease manifestations compared with the DENV-1 and DENV-3 infections.^43^ This finding was also comparable with a more recent study in Thailand which revealed higher proportions of DENV-2 and DENV-4 serotypes among cases with secondary compared with primary infection, while showing less pronounced differences between primary and secondary cases with DENV-1 and DENV-3 infections.^44^ In addition, a prospective study in Thailand conducted from 1994 to 2004 also reported that DENV-2 appeared to be associated with more severe disease compared with DENV-1.^41^

During the 13-year period, sustained switches in predominantly circulating DENV serotypes were detected about 2–6 months before the epidemics became apparent. A sustained change in the predominant serotype has served as an early warning of an impending surge in the number of dengue cases.^10,16,26^ Since 2013, the early warning system has been further supported by a statistical model projection. Together, the serotype and case-based early warning allow ramping up of preemptive vector control and community mobilization efforts well before the “traditional” dengue season.^23^ The National Environment Agency has been rolling out an annual dengue awareness and control campaign. Although the campaign in 2005 was launched in September^9^ during the peak of the epidemic in the first cycle, it was launched in April 2013 ahead of the peak in the second cycle. This could have contributed to the earlier peak in E-week 25 (June) and subsiding earlier in 2013, when compared with 2005 (peak in E-week 36, September).

The higher IR of indigenous cases living in landed properties compared with public and private apartments coincides with the consistently highest trend of *Aedes* HI for landed properties.^9,26^ This observation is also supported by two national health surveys in 2004^45^ and 2010^46^ which showed that DENV seroprevalence in adult Singapore residents was highest among those living in landed properties. The environment of landed properties is more likely to promote mosquito breeding because of the presence of front porch gardens, relatively larger spaces, and often large numbers of artificial containers. The marked and steady increase in the proportion and IR of dengue among those staying in private apartments was evident since 2013. This could be because of the sharp increase in the number of private apartments (38% increase from 2010 to 2017),^47^ dense development, and their colocation with densely populated public apartments. These factors would result in a significantly higher proportion of Singapore residents living in private apartments over the years (11.5% in 2010 to 13.9% in 2015)^48^ and higher population density. Along with the development, we observed a gradual geographical expansion of *Ae. aegypti*, despite a low HI and low *Aedes* mosquito density. The types of breeding habitats of *Aedes* mosquitoes remained consistent throughout the 13-year period.

Singapore’s integrated dengue control program has evolved over the 13-year period to address the challenges of the dynamic dengue situation. Enhancements include more comprehensive epidemiological investigations of cases, which helped to establish epidemiological links between cases, thus contributing to the increase in the proportion of dengue cases associated with clusters (30.9% in 2003 to 58.2% in 2016). Laboratory diagnostic testing has been widely available since 2008, when the use of rapid NS1 test kits was promoted for early diagnosis of cases, and improved case notification rate. To encourage residents to take ownership and eliminate mosquito breeding habitats in homes, a mobile application for greater public reach (myENV) and a color-coded alert banner system were launched in 2013 to keep residents informed about active dengue clusters in their respective localities. Together, these could have played a significant role in the increasing number of notified cases over the past decades since the 1980s, which would help explain the incongruence between the increasing IR of dengue and yet stable seroprevalence among Singapore residents.^28^ To better prioritize resources, data analytics is progressively being used to aid decision-making, such as adopting a risk assessment approach to target premises at higher risk of DENV transmission.

There are a few limitations to our study. The case surveillance system does not capture the actual number of DENV infections and the proportion of laboratory-confirmed cases reported to the MOH has evolved through the years as convenient and early laboratory diagnostic tools have been made readily available. The commonly used NS1 antigen test is also known to yield low sensitivity among secondary dengue infections.^49–51^ Together, the two limitations have certainly led to an underestimation of dengue cases in Singapore. Nevertheless, these limitations are expected to have little impact on the trends and patterns observed in this study.

## CONCLUSION

In summary, Singapore experienced an oscillation of DENV-1 and DENV-2 epidemics during the 13-year period from 2004 to 2016. Older adults appeared to be more affected by DENV-2 than DENV-1. Although there was no major shift in the types of breeding habitats of the primary dengue vector, *Ae. aegypti*, its distribution has expanded across the island. Singapore continues to be under the threat of dengue epidemics.

## Supplementary Material

Supplemental Figures.
