# Supplementary material for: Dengue in Singapore from 2004 to 2016: Cyclical Epidemic Patterns Dominated by Serotypes 1 and 2
Source: Am J Trop Med Hyg. 2018 May 29;99(1):204–10. doi: 10.4269/ajtmh.17-0819 (PMC6085773; doi:10.4269/ajtmh.17-0819)
Supplement: Supplementary file 1 [file tpmd170819.SD1.pdf]

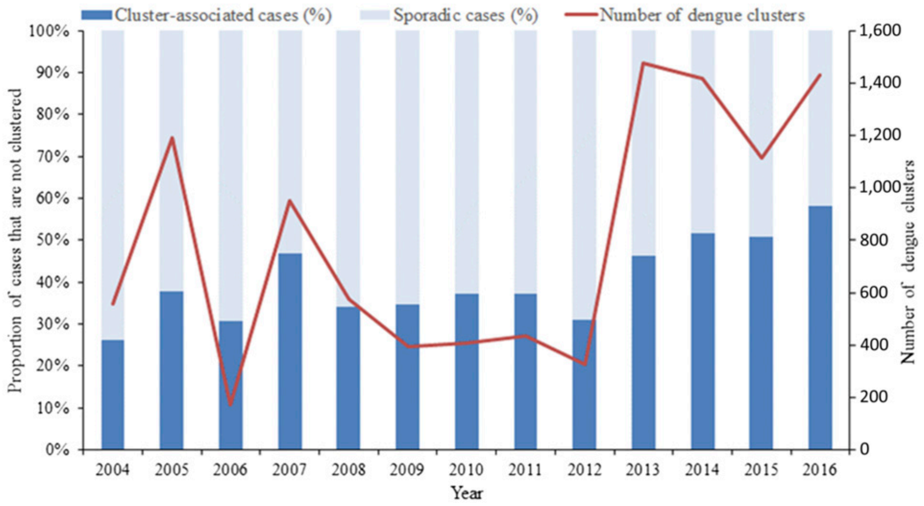

Supplemental Figure 1. Annual number of clustered and proportion of clustered and sporadic cases, 2004–2016.

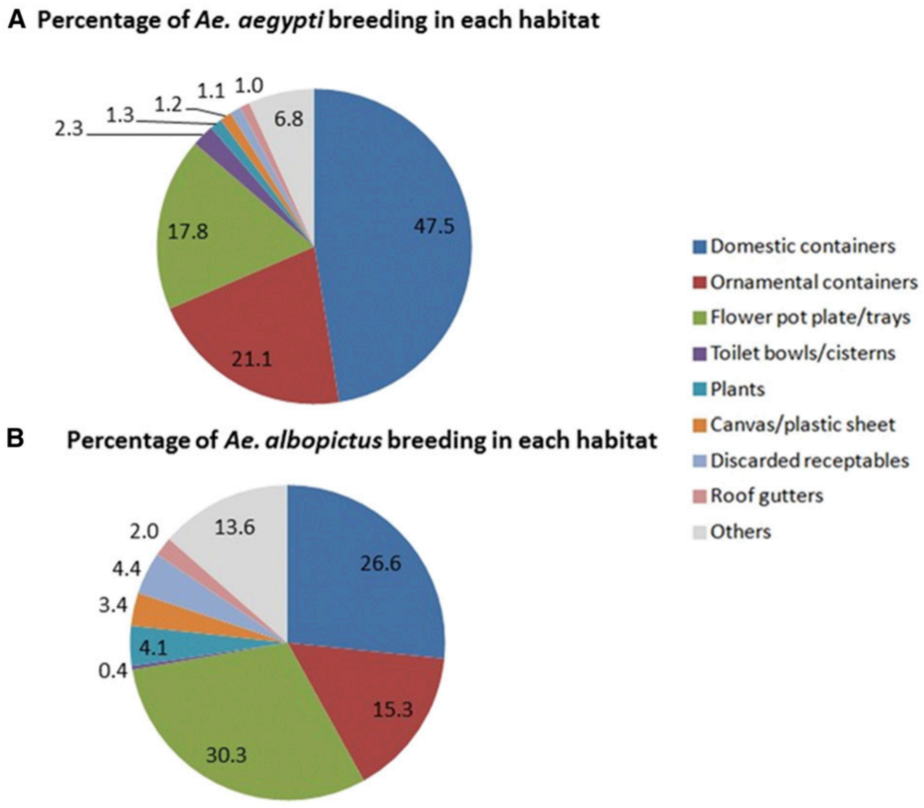

Supplemental Figure 2. Percentage of *Ae. aegypti* and *Ae. albopictus* breeding detected in the top eight breeding habitats, 2004–2016. (**A**) Percentage of *Ae. aegypti* breeding in each habitat. (**B**) Percentage of *Ae. albopictus* breeding in each habitat.

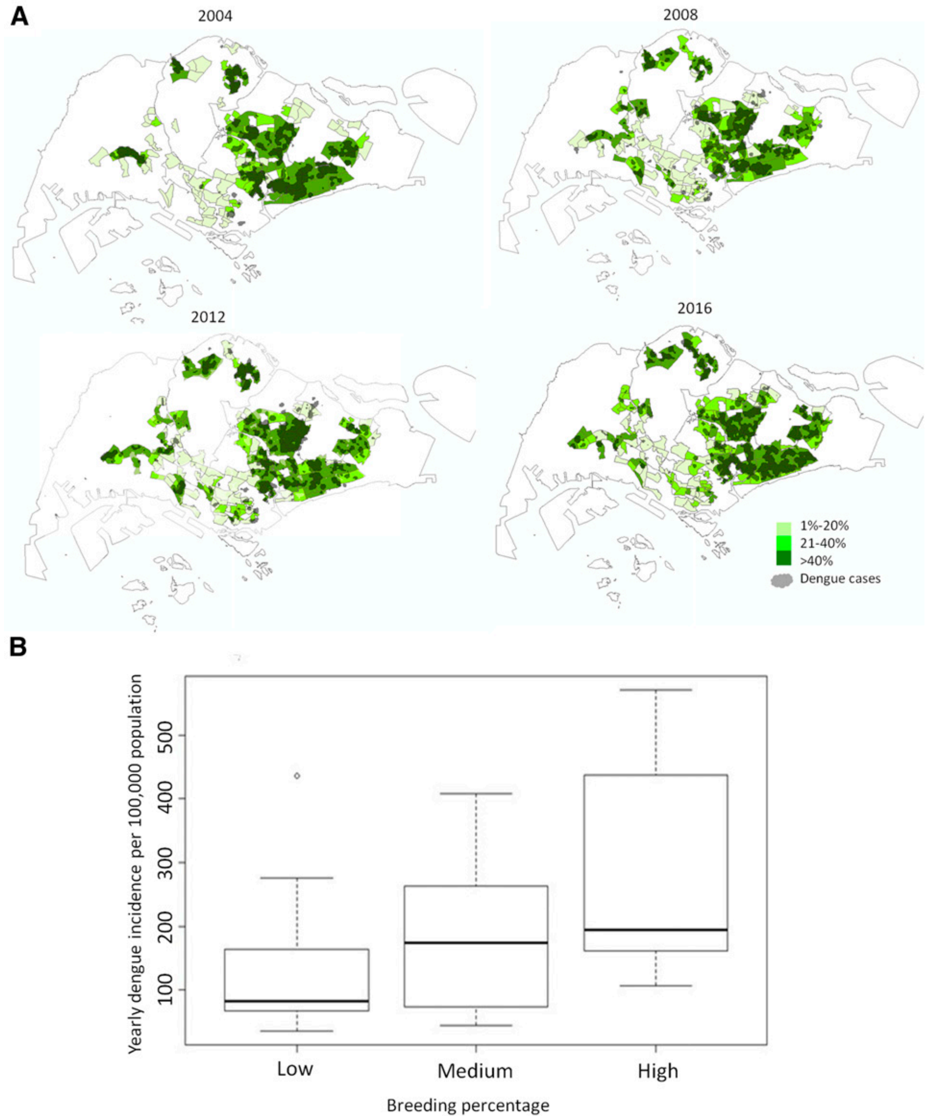

Supplemental Figure 3. Spatial distribution of *Ae. aegypti* breeding percentage (BP) areas overlaid with high dengue incidences: 2004, 2008, 2012, and 2016. Case density values of more than 75th percentile, using the quartile classification method, for each year are displayed in the darker green tone (year 2004: > 52 cases/km^2, year 2008: > 34 cases/km^2, year 2012: > 22 cases/km^2, and year 2016: > 62 cases/km^2). Boxplot indicates that dengue incidence is significantly higher in areas with high BP than areas with low BP (P value < 0.05).
